# Exploring the Applicability of General Dietary Recommendations for People Affected by Obesity

**DOI:** 10.3390/nu15071604

**Published:** 2023-03-25

**Authors:** Matthias Marsall, Gerrit Engelmann, Martin Teufel, Alexander Bäuerle

**Affiliations:** 1 Institute for Patient Safety (IfPS), University Hospital Bonn, 53127 Bonn, Germany; 2 Clinic for Psychosomatic Medicine and Psychotherapy, University of Duisburg-Essen, LVR-University Hospital Essen, 45147 Essen, Germany; 3 Center for Translational Neuro- and Behavioral Sciences (C-TNBS), University of Duisburg-Essen, 45147 Essen, Germany

**Keywords:** obesity, dietary recommendations, dietary guidelines, dietary behavior, assessment

## Abstract

(1) Obesity has emerged as a major public health challenge with increasing prevalence globally. The General Dietary Behavior Inventory (GDBI) was developed based on official dietary recommendations. However, little is known about whether general dietary recommendations also apply to people affected by obesity and whether the GDBI can be used appropriately. (2) A cross-sectional study was conducted. A total of 458 people meeting the inclusion criteria participated in the study. The assessment consisted of the GDBI and behavioral, dietary, and health-related variables. We used descriptive analysis to examine the item characteristics of the GDBI and inferential statistics to investigate the associations between the GDBI score and behavioral, dietary, and health-related outcomes. (3) Several items of the GDBI were concerned by ceiling effects. A higher GDBI score (indicating a higher adherence to dietary recommendations) was related to higher age, higher nutrition knowledge, more restrained eating behavior, lower impulsivity, and higher body mass index. There were no associations between the GDBI score and reported physical and mental health or quality of life. (4) The GDBI showed inconsistent relationships with the study outcomes. General dietary recommendations do not appear to be applicable to people with obesity. Hence, there is an urgent need for specific recommendations and subsequent assessments of behavioral adherence for people affected by obesity.

## 1. Introduction

In the global population, an increased prevalence of obesity has been observed for many years, which has already been focused upon by national and international scientific studies [1,2]. Using data from 2016, the World Health Organization (WHO) showed that more than 1.9 billion adults worldwide were overweight, and 650 million people were obese, which corresponds to a threefold increase since 1975 [3]. The consequences of obesity on people’s health have been considered and links between obesity and comorbidities have been identified; for instance, obesity is associated with diseases such as cancer [4], diabetes mellitus [5], and cardiovascular diseases [6]. In addition, the comorbidity rate with mental illnesses, such as depression, post-traumatic stress disorder, and substance abuse, is high [7,8]. People affected by obesity are also more vulnerable to infection with viral diseases such as severe acute respiratory syndrome coronavirus-2 (SARS-CoV-2), as well as to a more severe progression of the disease and increased risk of hospitalization and ICU admission [9,10]. Moreover, in the inpatient care of people with obesity, complications such as pressure injuries as well as perioperative complications, such as intubation difficulties, occur more frequently [11,12,13]. Considering the data from the Global Burden of Disease study from 1990 to 2017, overweight and obesity led to 70.7 million disease-adjusted life years (DALY) in females and 77.0 million in males; moreover, approximately 4.7 million deaths are associated with overweight and obesity worldwide [14].

Different studies looked at the medical care costs of obesity in different countries and found that the resulting costs for public health systems are considerable [15,16,17]. It has been shown that up to 4.7 percent of annual health care costs and up to 2.8 percent of annual hospital care costs in European countries are related to the treatment of obesity and its associated comorbidities [18,19]. Besides the direct costs of obesity, socioeconomic consequences such as sick leave, the need for long-term care or rehabilitation, early retirement, and compensation for unemployment of affected individuals are linked with obesity [16]. In summary, obesity is an increasing public health problem emerging worldwide that has a strong impact not only on individuals’ health status, but also on the public health system and national economies in many countries [20,21].

The etiology of obesity is manifold: it may be caused by genetic dispositions, monogenic disorders, neurologic, endocrine, and psychological disorders, and can also be triggered by the use of medication [22]. However, a strong connection between obesity and dietary behavior is evident: a healthy or an unhealthy dietary behavior is one of the most important factors influencing an individual’s weight [23,24,25]. The WHO, along with national institutions such as the German Nutrition Society (DGE), give specific recommendations on a balanced diet to promote one’s own health status and to reduce the risk of obesity [26,27]. These recommendations are easy to understand and make indications for a healthy dietary behavior for the average adult population. Different studies have already shown that obesity is associated with maladaptive dietary behavior that differs from international dietary recommendations [28,29,30].

One instrument that has been developed to assess adherence to the dietary recommendations of the WHO and the DGE is the General Dietary Behavior Inventory (GDBI) [31]. The GDBI was developed based on the specific dietary recommendations, and higher scores of the GDBI indicate higher adherence to those recommendations. Previous studies have shown that higher GDBI scores are associated with higher nutrition knowledge, lower body weight, and lower body mass index (BMI), as well as better self-rated physical and mental health and higher life satisfaction [31,32]. However, the GDBI was validated using general population samples [31,32]. An examination of whether dietary recommendations, as measured by the GDBI, also apply to people affected by severe obesity has not yet been studied.

This study aims to critically examine whether the dietary recommendations of the WHO and DGE, operationalized by the GDBI, are related to behavioral, nutrition-related, and health-related outcomes in individuals affected by obesity. Specifically, we expected that people who indicate that they are more adherent to the mentioned recommendations are less impulsive, have lower scores of eating disorder psychopathology, lower BMI, better nutrition knowledge, and report better health-related outcomes.

## 2. Materials and Methods

### 2.1. Study Design and Participants

A cross-sectional study in a German-speaking sample, using an online survey, was conducted. The assessment was carried out via the web-based survey tool Unipark (Tivian XI GmbH). The data collection took place between March 2022 and May 2022. Participants were recruited in (over)weight-related social media groups. We used an online flyer with specific information about the study. The flyer was posted in various social media groups (e.g., Facebook). We selected groups in which members shared information about diet, eating behaviors, obesity, and therapeutic options (e.g., bariatric surgery). There was no compensation offered for participation in this study and the participants could stop the survey at any time without negative implications. The inclusion criteria were: age of 18 years or older, BMI between 30 and 70, internet access to take part, and fluency in German. Moreover, we only included individuals who had not previously undergone bariatric surgery, as dietary recommendations, and dietary habits differ significantly for persons after bariatric surgery [33]. Further, we only included participants who completed all items of the GDBI. 458 people meeting the inclusion criteria participated in the study. After excluding 23 cases since they were identified as outliers in terms of questionnaire completion time, the final sample was N = 435. The Ethics Committee of the Medical Faculty of the University of Duisburg-Essen approved the conduction of the study (20-9718-BO).

### 2.2. Measurements

#### 2.2.1. Sociodemographic and Medical Characteristics

Sociodemographic and medical data, e.g., age (entered as actual age by free text input), sex (female, male, or diverse), educational level (no school certificate, secondary school certificate, completed vocational training, university entrance qualification, or university degree), marital status (married, not married, but in partnership, single, or other), body weight (in kilograms), and body height (in centimeters) were assessed. Furthermore, information regarding general nutrition behavior (omnivore diet, vegetarian diet, vegan diet, or other diet) and whether participants suffered from food intolerances (yes vs. no) were collected.

#### 2.2.2. Dietary Behavior

To assess dietary behavior of the sample, the “General Dietary Behavior Inventory” (GDBI) was implemented [31]. The items of the GDBI are included in Table S1. The instrument is based on the general dietary recommendations provided by the WHO [27] and the DGE [26,34,35]. The assessment comprises 16 items on a five-point bipolar scale. The response options are based on a semantic differential, which demonstrates concrete opposite dietary behaviors on the scale ends. A sample item is “I do not eat sweets (e.g., chocolate, cookies, pastries)” compared to “I eat sweets (e.g., chocolate, cookies, pastries) every day”. Cronbach’s alpha and the examination of factorial structures for the GDBI were not indicated because dietary behavior reflects different, uncorrelated behaviors. Therefore, the underlying measurement model of the GDBI is a formative rather than a reflective measurement model that cannot be subject to classic construct validation via factor analysis and the evaluation of reliability in terms of internal consistency [36]. The GDBI score was calculated as a sum of all items considering recoding of two reversed items (see Table S1). A higher GDBI score indicated more adherence to the respective recommendations.

#### 2.2.3. Behavioral and Nutrition-Related Constructs

To examine the general nutrition knowledge of the participants, we applied the nutrition knowledge questionnaire [37]. The 20-item instrument was answered on a 3-point scale (right/wrong/do not know). The total sum score indicated the extent of the participants’ nutrition knowledge. Ratings of “do not know” were treated as wrong. Higher scores indicated more comprehensive nutrition knowledge. The Cronbach’s alpha in this sample was 0.70.

To measure eating disorder psychopathology in the sample, the Eating Disorder Examination Questionnaire (EDE-Q8; eight-item version) was applied. The EDE-Q8 is a shortened version of the EDE-Q, which includes four subscales: restraint (Cronbach’s alpha = 0.70), eating concern (Cronbach’s alpha = 0.53), shape concern (Cronbach’s alpha = 0.80), and weight concern (Cronbach’s alpha = 0.71) [38]. As eating concern reached insufficient reliability, this subscale was excluded from the analyses. All items were assessed on seven-point Likert scales.

Furthermore, the eight-item Impulsive Behavior—8 Scale [39] was used to measure impulsivity. This self-report instrument assessed impulsive thoughts and behaviors that do not specifically relate to nutrition. The items were assessed via five-point Likert scales. The Cronbach’s alpha in this sample was 0.68.

#### 2.2.4. Health-Related Outcomes

Several health-related outcomes were examined (BMI, quality of life, physical health, and mental health). The BMI was calculated from the reported body weight and body height. Quality of life was assessed on a 5-point Likert scale from 1 = “not satisfied at all” to 5 = “totally satisfied” [40]. Physical health and mental health were each assessed on an 11-point Likert scale from 1 = “very bad health” to 11 = “very good health”. These items were validly used in previous studies [31,41].

### 2.3. Statistical Analyses

R and RStudio, as well as several packages (car, foreign, Hmisc, psych, and tidyverse) were used for all statistical analyses [42,43,44,45,46,47,48]. The BMI of all participants was calculated by considering body weight and body height. Furthermore, the sample was stratified for BMI categories (obesity grade 1: BMI 30 to <35; obesity grade 2: BMI 35 to <40; obesity grade 3: BMI >40). Descriptive statistics (e.g., mean and sum scores) were used to report the characteristics of the study sample (e.g., general nutrition knowledge scores) and GDBI characteristics (e.g., item response distributions). Group differences were examined using t-tests and analysis of variance (ANOVA). Pearson correlations were used to investigate interrelations. The relationships between the GDBI score and obesity grades were examined using logistic regression with odds ratio (OR) and 0.95 confidence intervals (CI). Sociodemographic covariates were applied in the additional regression analyses. Statistical significance was set to 5% (*p* = 0.05). Scale reliabilities were computed as Cronbach’s alpha.

## 3. Results

### 3.1. Study Sample Description

The mean age of the participants was 41.5 years (SD = 11.1). Table 1 shows the sociodemographic sample characteristics.

The average nutrition knowledge score was M = 12.8 points (SD = 3.7, range = 1–20). Participants rated their quality of life, on average, at 3.22 (SD = 0.9). Physical health was 5.86 (SD = 2.1) and mental health was 6.76 (SD = 2.5) on average. Impulsivity had a mean score of 3.01 (SD = 0.6). The EDE-Q subscales had the following means: restraint M = 4.51, SD = 1.6; shape concern M = 6.12, SD = 1.3; weight concern M = 6.21, SD = 1.2.

### 3.2. General Dietary Behavior Inventory—Descriptive Statistics and Relationships to Sociodemographic Variables

The item statistics of the sixteen GDBI items are shown in Table 2.

As shown in Table 2, several dietary behaviors were indicated as being followed by most participants (>50% of the participants indicating 4 or 5 on the scale). These items were: item 2 (all meals contain animal products), item 7 (always use vegetable oils), item 9 (never eat fast-food products), item 10 (never drink beverages containing sugar/sweeteners), item 12 (drink at least 1.5 liters of unsweetened fluid daily), and item 13 (never drink alcoholic beverages).

Within the possible range of 16–80 points in the GDBI score, the average score was 54.40 (SD = 7.8, median = 55). The lowest score was 30 and the highest score was 80. The GDBI score was used to investigate interrelations with sociodemographic and nutrition-related characteristics. The results are summarized in Table 3. We excluded the following subgroups due to small subgroup sample sizes: educational background: no school certificate and marital status: other. Further, we did not perform a significance test regarding general nutrition behavior as all groups except omnivores had small subgroup sample sizes.

A higher GDBI score was only significantly related to higher age, but unrelated to all other sociodemographic characteristics.

### 3.3. Associations between Dietary Behavior and Behavioral-, Nutrition-, and Health-Related Outcomes

We investigated the associations between the adherence to the dietary recommendations, as measured by the GDBI, and behavioral-, nutrition-, and health-related outcomes. The results of these analyses are displayed in Table 4.

Results revealed significant associations of GDBI score with nutrition knowledge, restrained eating behavior (EDE-Q-restrained), impulsivity, and BMI. Subsequently, we investigated the associations between the outcome variables and sociodemographic variables. We performed regression analyses to examine the relationships between the GDBI score and outcome variables considering sociodemographic variables, which were significantly related to the respective outcomes, as covariates. The results of these analyses are summarized in Table S2. The results of these analyses largely confirmed the findings of our initial analysis. However, when controlling for the covariates of age, educational background, and marital status, there was no longer a significant relationship between GDBI score and nutrition knowledge (B = 0.03, *p* = 0.20). Furthermore, there was no longer a significant relationship between the GDBI score and BMI when controlling for the covariates of age, sex, and educational background (B = 0.12, *p* = 0.054).

Further, we tested the group differences regarding the GDBI score and obesity grades. The results are summarized in Table 5.

The participants with higher obesity grades had slightly, but insignificantly, higher GDBI scores. When controlling for the covariates of age, sex, and educational background, the association between the GDBI score and obesity grades remained insignificant (B = 0.02, OR = 1.02, CI = 0.98–1.06, *p* = 0.31).

## 4. Discussion

### 4.1. Principal Findings

Obesity, which is also increasingly occurring in children and adolescents, and its impact on individuals and public health have emerged as growing problems over the past decades internationally [49,50]. People affected by obesity experience limitations in their daily life, such as pain, physical and mental impairments, social stigma, and work-related discrimination [21,51,52]. Furthermore, the financial costs for health care systems are considerable [17]. Since dietary behavior is one of the strongest predictors of obesity, it seems particularly important to evaluate dietary behavior in an obese sample and the assess the adherence to specific dietary recommendations. The present study is, to our knowledge, the first investigation to examine whether official dietary recommendations are appropriate guidelines for people affected by obesity. Our analyses showed differential and partly unexpected results, which are discussed below.

First, the examination of the GDBI characteristics in the study sample has shown that there were several dietary behaviors for which most participants indicated that they follow. This was reasonably unexpected, as some of the related behaviors (e.g., the consumption of fast food or sweetened beverages) are associated with higher body weights and obesity [53,54]. However, this may also reflect that our study sample consisted predominantly of individuals who made an effort to follow general dietary recommendations, but were still suffering from obesity. This is consistent with previous findings indicating that long-term weight loss in people affected by severe obesity is unlikely to be achieved through lifestyle changes, but is often subject to weight regain [55]. Frequently, bariatric surgery is the last option for successful long-term weight loss in people with obesity [56,57]. Nevertheless, the ceiling effects of several GDBI items indicated that it is questionable whether a self-assessment can contribute to the valid measurement of dietary behaviors in people with obesity.

In line with our expectation, higher GDBI scores were significantly related to better nutrition knowledge. On the one hand, this result corroborates previous study findings indicating that people who have a better knowledge about nutrition and food eat more healthily [31,32,58]. On the other hand, when sociodemographic covariates were controlled for, there was no longer a significant relationship between nutrition knowledge and the GDBI scores. Rather, a relevant influence of educational background was found to explain a significant proportion of the variance in nutrition knowledge (see Table S2), which is in line with previous research findings [59]. This finding contradicts our assumption that people with better nutrition knowledge are more likely to adhere to dietary recommendations, as measured by the GDBI.

Regarding eating disorder psychopathology, a higher compliance with the dietary recommendations was highly related to more restrained eating behavior (EDE-Q restraint subscale). Nevertheless, there were no associations between the GDBI score and concerns regarding body weight (EDE-Q weight concern) and body shape (EDE-Q shape concern). These results imply that persons who follow official dietary guidelines exhibit more restrained eating behaviors, regardless of the cognitive aspects of weight and shape concerns. 

With regard to impulsivity (what was not assessed directly in terms of nutrition), this study confirms a significant negative association between impulsivity and adherence to dietary recommendations, consistent with previous study findings [60]. Taking the significant relationship between higher GDBI scores and lower impulsivity and more restrained eating behaviors together, we conclude that people who are less adherent to dietary recommendations, as measured by the GDBI, behave impulsively more often. Concurrently, these individuals show a lower degree of compulsive eating behaviors. Overall, these results suggest that individuals with higher adherence to dietary recommendations are more restrictive in their food intake and less impulsive in general. These findings resonate well with the previous research regarding the relationship between higher impulsivity, and higher likelihood of eating disorders [61,62].

Regarding the health-related outcomes of the GDBI, our results were in contradiction to previous research on the association of GDBI score and health-related outcomes and BMI [31,32]. Interestingly, in this study, there was no association between GDBI scores and physical and mental health or quality of life. These results remained consistent after the inclusion of sociodemographic variables in the additional analyses. Even more unexpected was the positive relationship found between GDBI scores and BMI, indicating that people who were more compliant with the dietary guidelines had a higher BMI. However, this effect was no longer evident when controlling for relevant sociodemographic variables. This underpins the finding that there was no significant association between adherence to dietary recommendations, as assessed by the GDBI, and obesity grade. However, it should be noted that the GDBI does not seem to be capable of distinguishing between people with and without obesity, since the GDBI score in this study was comparable to scores from previous studies with heterogeneous samples [31,32]. This implies that people with obesity report a similar level of adherence to dietary recommendations as people in the general population. This result could either reflect a misconception of the dietary recommendations of the GDBI in the context of people affected by obesity, or that there were people in this sample who tended to answer the questionnaire items incorrectly in the sense of dissonance reduction or social desirability [63,64]. However, we consider the influence of social desirability in the response to our questionnaire to be rather low, since it was a completely anonymous survey.

To sum up, the assessment of dietary behavior in people affected by obesity is a complex and yet unsolved issue. The results of our analyses point out that dietary recommendations tailored to the general population may not be applicable for people affected by obesity. Dietary behaviors and related outcomes can vary widely among individuals and population groups. We assume that the general dietary recommendations do not cover the needs of people affected by obesity. Therefore, population-specific dietary recommendations should be developed to help people affected by obesity to reflect on and change their dietary behavior. There are examples of such specific dietary recommendations for patients after bariatric surgery: Sherf Dagan and colleagues summarized dietary recommendations in a review article [33], and Moizé and colleagues developed the nutritional pyramid for patients after bariatric surgery [65].

Consequently, the GDBI, as a valid instrument for measuring dietary behavior in the general population, should not be used in the assessment of dietary behavior in people with obesity. One reason could be that the GDBI was designed to measure dietary behavior in a qualitative way and does not take into account the quantities and frequencies of eating. We argue that the measurement of dietary behavior in people affected by obesity should be either assessed in a complementary way that combines the GDBI with food frequency questionnaires [66,67], or by developing a new instrument that covers the situation and circumstances of this population. Other approaches, such as video analyses [68] or sensing technology [69], should also be considered as a substitute for self-reported questionnaires. Therefore, future studies should engage with the conceptualization and valid measurement of dietary behavior in people affected by obesity.

### 4.2. Limitations

The results of the present study should be interpreted considering the following limitations. Due to the cross-sectional study design, no conclusion regarding causality can be drawn from the data. Furthermore, due to the online recruitment, a potential selection bias cannot be ruled out. All data were self-reported and might be biased. The studys’ sample approach was convenience sampling, resulting in a possible sampling bias. Consequently, the generalizability of the study results may be limited, as the study sample consisted mainly of female participants and almost entirely of people with an omnivorous diet. Even though we controlled for the influences of sociodemographic variables in the additional analyses, the generalizability of the study results, particularly with respect to sex, remains limited. Moreover, the majority of our sample had a BMI above 40 (obesity grade 3), which does not reflect a representative sample of people affected by obesity.

## 5. Conclusions

Dietary recommendations developed for the general population probably do not cover the individual circumstances of people who are affected by obesity. Population-specific dietary behavior guidelines should be developed. Moreover, the assessment of the dietary behaviors in people affected by obesity should not be based solely on self-reported measurement, or at least be complementarily assessed using multiple instruments to consider a set of different aspects of dietary behaviors.

## Figures and Tables

**Table 1:** Sociodemographic sample characteristics.

Characteristic	*N*	%	Missing (%)
**Sex**			103 (23.7)
Female	303	69.7	
Male	29	6.7	
**Educational background**			101 (23.2)
No school certificate	3	0.7	
Secondary school certificate	97	22.3	
Completed vocational training	128	29.4	
University entrance qualification	36	8.3	
University degree	70	16.1	
**Marital status**			101 (23.2)
Married	173	39.8	
Not married, in partnership	84	19.3	
Single	68	15.6	
Other	9	2.1	
**General nutrition behavior**			99 (22.8)
Omnivore	315	72.4	
Vegetarian	6	1.4	
Vegan	4	0.9	
Other	11	2.5	
**Food intolerance**			99 (22.8)
Yes	60	13.8	
No	276	63.4	
**BMI (Mean = 43.2, SD = 8.3)**			100 (23.0)
Obesity grade 1 (BMI 30 to <35)	62	14.3	
Obesity grade 2 (BMI 35 to <40)	65	14.9	
Obesity grade 3 (BMI above 40)	208	47.8	

BMI: body mass index

**Table 2:** Descriptive item statistics of the GDBI items.

Item	Mean	SD	Skew	Response Distribution in Percent				
				1	2	3	4	5
gdbi1	3.44	1.10	0.00	6	3.9	57	6.4	26.7
gdbi2	4.03	1.06	−0.54	1.8	2.1	37.2	8.7	50.1
gdbi3	3.63	1.08	−0.06	3.4	3.7	51.7	8.7	32.4
gdbi4	3.07	1.10	0.23	7.6	18.9	48.3	9.4	15.9
gdbi5	3.04	0.81	0.49	2.3	16.3	63.4	10.6	7.4
gdbi6	2.96	1.31	−0.01	20.5	8.5	43	10.6	17.5
gdbi7	3.81	1.11	−0.41	3.4	3.9	40	13.3	39.3
gdbi8	2.68	1.24	0.05	26.9	9.2	41.8	13.6	8.5
gdbi9	3.69	0.93	−0.13	2.3	1.4	45.7	26.2	24.4
gdbi10	3.49	1.45	−0.51	16.8	6	24.6	17.2	35.4
gdbi11	3.23	1.45	−0.19	18.9	9.7	30.6	11.3	29.7
gdbi12	3.64	1.52	−0.63	16.6	6.4	20.7	9.4	46.9
gdbi13	4.52	0.93	−2.03	2.3	1.6	11.7	10.1	74.3
gdbi14	3.18	1.19	−0.07	11.7	8.7	49	11.3	19.3
gdbi15	2.69	1.44	0.30	30.3	14.3	29.4	7.8	18.2
gdbi16	3.30	1.32	−0.22	14	7.1	40.9	10.6	27.4

**Table 3:** Interrelations between the GDBI score and sociodemographic characteristics.

Characteristic	GDBI Score		Test Statistic	*p*
	Mean	SD		
**Age**	-	-	r = 0.29	<0.001
**Sex**				
Female	54.93	7.64	t = 0.50 (df = 330)	0.61
Male	54.17	8.64		
**Educational background**				
Secondary school certificate	55.43	8.51	F = 1.08 (df = 3, 327)	0.36
Completed vocational training	53.98	7.92		
University entrance qualification	56.17	7.02		
University degree	54.90	6.48		
**Marital status**				
Married	54.90	7.34	F = 0.19 (df = 2, 322)	0.83
Not married, in partnership	55.00	8.53		
Single	54.26	7.80		
**Food intolerance**				
Yes	54.45	7.80	t = −1.88 (df = 334)	0.06
No	56.50	7.14		

**Table 4:** Interrelations between the GDBI score and outcome variables.

Outcome Variable	Pearson Correlation Coefficient
**Behavioral- and nutrition-related outcomes**	
Nutrition knowledge	0.14 ***
EDE-Q—restraint	0.36 ***
EDE-Q—shape concern	−0.04
EDE-Q—weight concern	0.01
Impulsivity	−0.31 ***
**Health-related outcomes**	
BMI	0.15 **
Quality of life	−0.04
Physical health	−0.05
Mental health	0.04

** *p* < 0.01; *** *p* < 0.001. EDE-Q: Eating Disorder Examination Questionnaire.

**Table 5:** Obesity grades and GDBI scores.

Obesity Grade	GDBI Score		Coefficient B; OR (CI)	*p*
	Mean	SD		
Grade 1—BMI 30 to <35	53.34	8.16	0.03; 1.03 (0.99–1.07)	0.09
Grade 2—BMI 35 to <40	54.05	7.39		
Grade 3—BMI above 40	55.55	7.60		

OR: odds ratio; CI: 95% confidence interval.

## Data Availability

The data presented in this study are available on reasonable request from the corresponding author.

## Supplementary Materials

The following supporting information can be downloaded at: https://www.mdpi.com/article/10.3390/nu15071604/s1. Table S1: Items of the GDBI. Table S2: Results of regression analyses examining the relationship between GDBI score and study outcome variables considering sociodemographic covariates.
