# Supplementary material for: Exploring the Applicability of General Dietary Recommendations for People Affected by Obesity
Source: Nutrients. 2023 Mar 25;15(7):1604. doi: 10.3390/nu15071604 (PMC10097167; doi:10.3390/nu15071604)
Supplement: Supplementary file 1 [file nutrients-15-01604-s001.zip › Table S1 items of the GDBI.pdf]

Table S1. Items of the General Dietary Behavior Inventory

The following statements are about your dietary behaviors.

Item	5<br>Like Behavior A	4	3	2	1<br>Like Behavior B
gdbi1	I eat different foods every day.				I eat the same foods every day.
gdbi2	My meals always include animal products (e.g., meat, fish, eggs, dairy products such as yogurt, cream, cheese).				My meals do not include animal products (e.g., meat, fish, eggs, dairy products such as yogurt, cream, cheese).
gdbi3	My meals always include plant-based products (e.g., vegetables, grain products, tofu products).				My meals do not include plant-based products (e.g., vegetables, grain products, tofu products).
gdbi4	I eat at least 2 servings of fruit daily.				I never eat fruit.
gdbi5	I eat at least 3 servings of vegetables daily.				I never eat vegetables.
gdbi6	I eat whole grain products (e.g., whole grain pasta, whole grain bread) instead of white flour products (e.g., "conventional" pasta, white bread/grain bread) every day.				I eat white flour products (e.g., "conventional" pasta, white bread/grain bread) instead of whole grain products (e.g., whole grain pasta, whole grain bread) every day.
gdbi7	I always consume vegetable fats (e.g., margarine and oils based on canola, olive, sunflower) instead of animal fats (butter, lard, etc.).				I always consume animal fats (butter, lard, etc.) instead of vegetable fats (e.g., margarine and oils based on olives, canola, sunflowers).
gdbi8	I do not eat sweets (e.g., chocolate, cookies, pastries).				I eat sweets (e.g., chocolate, cookies, pastries) every day.
gdbi9	I do not eat fast food and convenience foods (e.g., frozen pizza, microwave ready-to-eat meals).				I eat fast food and convenience foods (e.g., frozen pizza, microwave ready-to-eat meals) daily.
gdbi10	I do not drink sugary/sweetened beverages (e.g., juices, soda, sweetened coffee/tea).				I drink sugary/sweetened beverages (e.g., juices, lemonade, sweetened coffee/tea) daily.
gdbi11	I consume at least 1.5 liters of fluid daily (excluding alcoholic beverages or beverages containing sugar/sweeteners).				I consume less than 1.5 liters of fluid (excluding alcoholic beverages or beverages containing sugar/sweeteners) daily.
gdbi12(r)	When preparing meals, it is important to me that they are prepared quickly (e.g., quick frying, quick/spicy sautéing).				When preparing meals, it is important to me that they are prepared slowly (e.g., slow cooking/frying with medium heat).
gdbi13	When seasoning my meals, I make sure to use as little salt as possible.				I like to use a lot of salt when seasoning my food.
gdbi14(r)	I drink alcohol regularly (daily or even every weekend).				I don't drink alcohol or drink it very rarely (only on special occasions).
gdbi15	I prefer to eat in peace without being distracted by anything.				While eating, I like to occupy myself with other topics (e.g., watching TV, thinking about work, reading).
gdbi16	When taking my meals, I like to take my time.				I like to take my meals quickly.

Items are scored from 5 (=Like Behavior A) to 1 (=Like Behavior B), except for the two inverted items. The GDBI score is the sum of all items' scoring.
