# Supplementary material for: Exploring the Applicability of General Dietary Recommendations for People Affected by Obesity
Source: Nutrients. 2023 Mar 25;15(7):1604. doi: 10.3390/nu15071604 (PMC10097167; doi:10.3390/nu15071604)
Supplement: Supplementary file 1 [file nutrients-15-01604-s001.zip › Table S2 Additional analyses.pdf]

Table S2. Results of regression analyses examining the relationship between GDBI score and study outcome variables considering sociodemographic covariates.

	Dependent variables								
	Behavioral and nutrition-related outcomes					Health-related outcomes			
	Nutrition knowledge	EDE-Q - restraint	EDE-Q – shape concern	EDE-Q – weight concern	Impulsivity	BMI	Quality of life	Physical health	Mental health
Predictor	Coefficient B								
Age	0.04*	0.01	-	-	-	0.09*	-0.01**	-0.06***	-
Sex	-	-	-0.45*	-0.48*	-	4.40**	-	-	-
Educational background	0.60***	-	-	-	-	-1.01*	0.13**	0.32**	-
Marital status	-0.46*	-	-	-	-	-	-0.13*	-0.31*	-
General nutrition behavior	-	-	-	-0.22*	-	-	-	-	-
GDBI score	0.03	0.07***	-0.00	0.01	-0.02***	0.12	0.00	0.01	0.01

Notes. * $p < 0.05$; ** $p < 0.01$; *** $p < 0.001$. Food intolerance was not associated with any outcome variable and therefore not included in the table. Educational background, marital status, and general nutrition behavior were treated as continuous variables to facilitate interpretation of results. For each outcome, only those covariates that were significantly related to it were included in the regression.
